# Sex Differences Associated with Weekend Catch-Up Sleep and Waist-to-Height-Ratio among South Korean Adults Using Korea National Health and Nutrition Examination Survey 2016–2021 Data

**DOI:** 10.3390/healthcare11212889

**Published:** 2023-11-02

**Authors:** Seungwon Jung, Jin Young Nam

**Affiliations:** Department of Healthcare Management, Eulji University, Seongnam-si 13135, Republic of Korea; wjdtmd115@naver.com

**Keywords:** adult, obesity, waist-to-height ratio, weekend catch-up sleep, weekend supplementary sleep

## Abstract

The global surge in obesity rates is closely linked to the rise in sleep deprivation and prevalence of sleep disorders. This study aimed to investigate the association between weekend catch-up sleep (CUS) and obesity among Korean adults. Using multiple logistic regression analysis, we analyzed the data of 6790 adults aged >19 years obtained from the Korea National Health and Nutrition Examination Survey 2016–2021. In the subgroup analysis, we conducted multiple logistic regression analysis to determine the association between weekend CUS and obesity, stratified by sex. Women were significantly more likely to be obese than men (odds ratio (OR) = 0.53, 95% confidence interval (CI) = 0.46–0.61). Obesity was associated with 1 ≤ weekend CUS < 2 (OR = 0.86, 95% CI = 0.75–0.99) but not with weekend CUS ≤ 0. Compared to men, women had a lower obesity risk when engaging in weekend supplementary sleep that was 1 ≤ weekend CUS < 2 (OR = 0.78, 95% CI = 0.63–0.97). Our findings revealed that weekend CUS was associated with obesity. Our findings suggest that weekend CUS may offer a form of biological protection against obesity, and they contribute to a better understanding of this association and may serve as a basis for better obesity management.

## 1. Introduction

Obesity is characterized by the abnormal or excessive accumulation of fat in adipose tissue, which poses significant health risks [1]. It is a significant risk factor for cerebrovascular diseases, heart conditions, and lifestyle-related diseases, including diabetes, hypertension, and hyperlipidemia [2].

Obesity carries an elevated risk of various health conditions, including type 2 diabetes mellitus, fatty liver disease, osteoarthritis, Alzheimer’s disease, depression, musculoskeletal conditions, and multiple types of cancer, such as that of the breast, ovaries, prostate, liver, kidney, and colon [3]. It has substantial health impact on individuals, society, and the economy, and its global prevalence has rapidly increased worldwide [4]. In Korea, the prevalence of obesity has steadily risen to 32.8% in 2012; 34.8% in 2017; and 37.2% in 2021 [5]. Therefore, efforts to reduce obesity prevalence and analyze the risk factors for its prevention are essential.

Previous studies have investigated obesity risk factors, including low levels of physical activity [6], genetic factors [7], and stress-related unhealthy eating habits [8]. Additionally, studies have reported that increased sleep deprivation and sleep disorders are associated with changes in obesity rates [9]. Over the past 15 years, the average sleep duration among surveyed Koreans has significantly increased from 411.1 min to 434.5 min, primarily attributed to delayed wake-up times and weekend supplementary sleep [10]. This is accounted for in the recommended 7–9 h of sleep for adults, as suggested by the Center for Disease Control and Prevention [11,12,13]. However, the severity of sleep deprivation is still emerging, as it necessitates the consideration of individuals’ constitution, health conditions, and daily life patterns [4]. In modern society, the significance of adequate sleep cannot be overstated. Insufficient sleep duration has been shown to have adverse effects on emotional control and emotional stability [14]. Moreover, sleep deprivation serves as a factor that elevates the risk of obesity, underscoring the paramount importance of prioritizing sleep [15]. To compensate for weekday sleep deficits, individuals often resort to taking naps or increasing their sleep duration on weekends [4,16]. Previous research has highlighted a correlation between this phenomenon, known as weekend catch-up sleep (CUS), and obesity. Some studies have explored this connection while directly or indirectly considering chronic inflammatory conditions [16]. Furthermore, a nationwide cohort study conducted in Korea unveiled an association between average sleep duration and difference in sleep duration between weekdays and weekends in relation to obesity [4]. Notably, weekend supplementary sleep has also been linked to a reduced risk of high blood pressure [17]. However, it is essential to acknowledge that sleep duration varies significantly among different demographic groups, including sex, age, and race. A recent study focused on Korean adults found that sleep deprivation was linked to an increased risk of obesity [18]. Additionally, other study confirmed that as sleep duration increased, the risk of obesity decreased [2]. To date, many studies investigating the relationship between sleep and obesity have primarily relied on body mass index (BMI) and similar markers. Nevertheless, there is a paucity of research analyzing other obesity indicators. To prevent health problems in adulthood stemming from obesity, continued interest and research are needed on the consistency of evaluation results from various obesity assessment methods. When measuring obesity, since direct measurement is very difficult, indirect measurement and evaluation are performed [19]. Accordingly, various international obesity assessment methods, including body mass index (BMI), waist circumference (WC), and waist-to-height ratio (WHtR), are widely used in daily life [19]. Among these methods, BMI is a widely used measurement, but it has limitations as it only takes into account height and weight, which can lead to errors in results [20]. For instance, individuals with a high muscle content may have a seemingly elevated BMI due to their weight, even though this does not necessarily indicate excessive fat content. In addition, WC may have a higher risk of measurement errors, and if used as an obesity indicator, there may be a problem of overestimating or underestimating various obesity-related diseases among tall and short people with similar waist circumferences [21]. One study identified which of the BMI, WC, and WHtR obesity evaluation methods was more accurate, and among them, the WHtR obesity evaluation method was more consistent than the BMI and WC obesity evaluation methods [19], and regardless of gender, WHtR was more sensitive than BMI or WC when determining obesity [22]. Therefore, we use the WHtR, as it is a more explanatory indicator for abdominal fat accumulation than BMI [23,24,25]. The hypothesis of this study is that adequate weekend catch-up sleep will reduce the risk of obesity. The objectives of this study was to evaluate the relationship between weekend catch-up sleep and obesity, and to determine whether weekend catch-up sleep is associated with obesity. Our findings will provide important information on obesity prevention and management and contribute to understanding the role of weekend catch-up sleep in reducing the risk of obesity-related diseases.

## 2. Materials and Methods

### 2.1. Study Participants and Database Information

**Figure 1:**
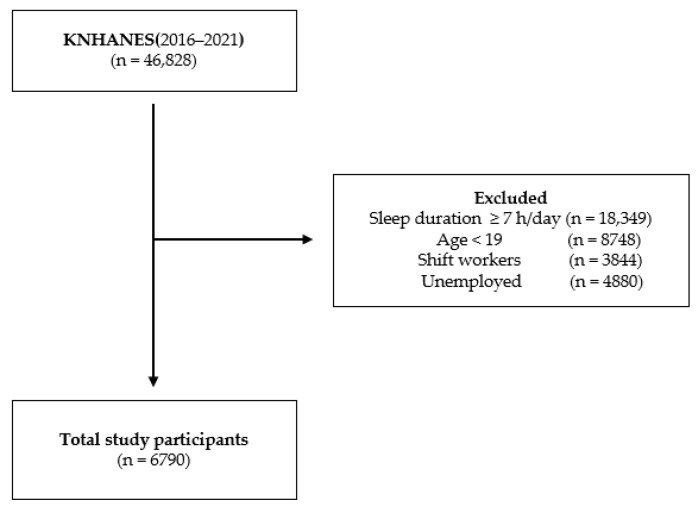
Flowchart of the sampling procedure.

Data is frequently collected from the Korea National Health and Nutrition Examination Survey (KNHANES), a national survey, to examine Koreans’ health and nutrition statuses. Since its inception in 2007, the survey is conducted annually. KNHANES employs multi-stage cluster sampling and targets non-institutionalized Korean citizens from the household registry. Furthermore, post-stratification is used to account for cross-sectional study designs. These surveys have been conducted to obtain reliable and representative national statistics on the health of the general population, including the health status and intake of food and nutrients; these data are used to set goals of the general plan for national health promotion, develop various health promotion programs, and inform health policies. The survey offers unbiased and consistent resources for calculating the prevalence of illnesses, conditions, and at-risk behaviors. The data in this study were collected from the seventh and eighth KNHANES conducted between 2016 and 2021. First, of the 46,828 individuals from the KNHANES VII-3, a total of 36,790 were included. In total, 40,038 individuals met the exclusion criteria. The non-weekend CUS group comprised 3476 participants, and the weekend CUS group comprised 3314 participants (0–1 h of CUS: 374, 1–2 h: 1281, ≥2 h: 1659 (Figure 1)).

### 2.2. Obesity

WHtR is a value obtained by dividing waist circumference by height [26] and is mainly used to evaluate abdominal obesity and health risk [27,28]. WHtR is advantageous in that it uses the same reference point regardless of age, gender, and race. It is also generalizable to other racial groups during international research [29]. According to previous studies, the WHtR obesity assessment method can effectively evaluate obesity in adults [30,31] and can compensate for concerns about height-related bias in various indicators that measure obesity [32]. In this study, WHtR was calculated as follows: waist (cm)/height (cm); the participants’ weight and height information were collected from KNHANES 2016–2021 during national health checkups. The anthropometric parameters were measured directly by experts. A WHtR value of ≥0.5 was defined as obesity, per the criteria from a previous study. A WHtR < 0.5 was considered normal [33].

### 2.3. Sleep Duration and Weekend CUS

Sleep duration was measured using responses to the following question: “How many hours do you usually sleep per day?” Using the average daily sleep length for the weekdays and weekends, the average sleep time was computed as follows: [average sleep duration per day 5 + average sleep duration per weekend 2]/7. Weekend CUS was divided into four groups and measured as the average weekend sleep length minus the average weekday sleep duration: ≤0, 0–1, 1–2, and ≥2 h. Non-CUS was defined as CUS ≤ 0 h [4].

### 2.4. Covariates

The following covariates were obtained using a standardized KNHANES questionnaire: age, sex, marital status, household income, educational level, region, occupation, smoking status, alcohol consumption, and physical activity. The participants were stratified by age into three groups: 19–39, 40–65, and >65 years. Marital status was categorized as single or married. Household income was divided into quartiles and categorized as low, low-moderate, moderate-high, and high. Educational level was categorized as elementary school, middle school, high school, college, or higher. Occupation was defined as white collar for managers, professionals, related workers, and office workers; pink collar for service and sales and simple labor workers; and blue collar for skilled agricultural, forestry, fishery workers, and workers in technical personnel/device machine operation and related functions. Regarding smoking status, participants were categorized into three groups: current smokers, ex-smokers, or never-smokers. Alcohol consumption was defined based on the frequency, with categories including no alcohol consumption, alcohol consumption once a week or less, and alcohol consumption more than twice a week. The physical activity of the study subjects was calculated using the Global Physical Activity Questionnaire (GPAQ) developed by the World Health Organization [34]. The GPAQ was translated into Korean in 2013 by the Korea Centers for Disease Control and Prevention, and has been used in the National Health and Nutrition Examination Survey since 2014 [35]. In this study, the amount of physical activity was converted into a continuous variable based on information from responses to questions in the GPAQ. Physical activity levels were classified as follows: moderate-intensity (physical activity > 150 min per week), high-intensity (physical activity > 75 min per week), and moderate-to-high intensity physical activity.

### 2.5. Statistical Analyses

We examined the association between weekend CUS and obesity based on sex. To evaluate baseline characteristics, a chi-squared test was used. The odds ratios (ORs) and 95% confidence intervals (CIs) for general characteristics and obesity were calculated using multiple logistic regression analyses. Multiple logistic regression analyses were performed on weekend CUS and obesity based on sex. Statistical significance was set at *p* < 0.05. SAS version 9.4 (SAS Institute Inc., Cary, NC, USA) was used for all the statistical analyses.

## 3. Results

The characteristics of the study population are summarized in Table 1. Of the 6790 participants, 3888 (57.3%) and 2902 (42.7%) were obese and of normal weight, respectively. The numbers of participants with weekend CUS hours of CUS ≤ 0, 0 < CUS < 1, 1 ≤ CUS < 2, and CUS ≥ 2 were 3476 (51.2%), 374 (5.5%), 1281 (18.9%), and 1659 (24.4%), respectively. The prevalence rates of obesity were 2231 (61.6%) and 1657 (52.5%) in men and women, respectively. The prevalence of obesity was 37.2% (*n* = 594), 59.5% (2544), and 81.7% (*n* = 750) among those aged 19–39, 40–65, and >65 years. Additionally, the prevalence of obesity was higher among the married (61.2%), lower income (73.5%), lower education (81.2%), rural (67.3%), and blue collar (64.5%) groups; the difference in the prevalence of obesity was significant between each group for all variables.

Table 2 shows the association between the general participant characteristics and obesity among South Korean adults. Women were not significantly more obese than men (OR = 0.53, 95% CI = 0.46–0.61). Moreover, obesity was associated with 1 ≤ CUS < 2 (OR = 0.86, 95% CI = 0.75–0.99), when compared with the reference group (Table 2). However, neither 0 < CUS < 1 (OR = 0.87, 95% CI = 0.69–1.09) nor CUS ≥ 2 (OR = 0.96, 95% CI = 0.84–1.09) were significantly associated with obesity.

**Figure 2:**
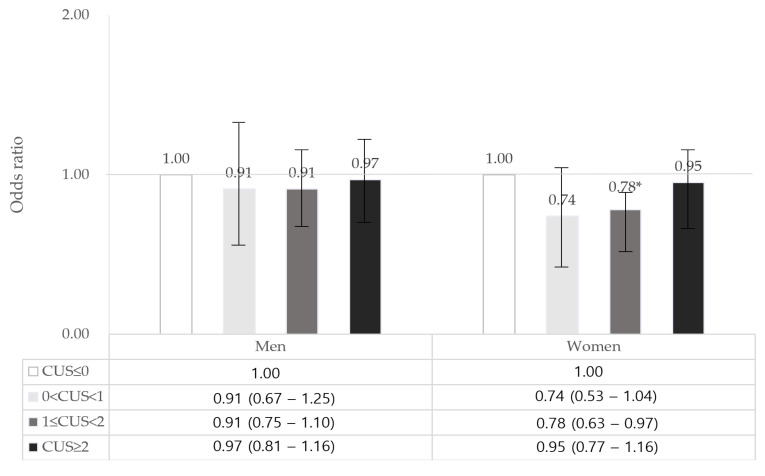
Association between weekend CUS and obesity according to sex. CUS, Catch-up sleep. Adjusted for age, sex, marital status, household income, educational level, occupation, region, smoking status, alcohol consumption, and physical activity; * *p* < 0.05.

In the subgroup analysis, we found sex differences in the association between weekend CUS and obesity among South Korean adults (Figure 2). Women who slept 1–2 h over the weekend showed a 22% lower risk of obesity (OR = 0.78, 95% CI = 0.63–0.97) (Supplementary Table S1) compared with that in the reference group. However, no significant association was observed between CUS and obesity in men.

## 4. Discussion

This study revealed that maintaining sufficient weekend CUS was linked to a lower occurrence of obesity. Furthermore, we observed a sex-specific difference in the relationship between weekend CUS and obesity. Several studies have consistently reported a connection between sleep duration and obesity. One earlier study noted that individuals who slept between 7 and 8 h per day were most likely to be at risk for obesity, and the longer one’s average sleep duration, the lower their obesity risk [4]. When the average sleep duration dropped below 6 h, the risk of obesity significantly increased, with a 1.2-fold higher risk associated with less than 6 h of sleep. In 2017, a study in Korean adults aged 18 to 70 years, further substantiated the link between insufficient sleep and increased total body fat that leads to obesity [18]. Furthermore, a study published in 2022 provided additional support for the association between sleep duration and obesity in both adult men and women. It revealed that the risk of obesity decreased as sleep duration increased [2]. Moreover, when specifically investigating the link between sleep duration and obesity based on sex, it was found that among women, obesity was exclusively associated with inadequate sleep duration [36]. Specifically, women who slept less than 5 h gained 1.14 kg more weight than those who slept for 7 h [37]. Numerous studies have elucidated the mechanisms that underlie the connection between sleep duration and obesity. Firstly, insufficient sleep impacts the secretion of appetite-regulating hormones like leptin and ghrelin, potentially contributing to obesity [15]. In an experiment, individuals who had less than 5 h of sleep had a higher BMI, an increased ghrelin concentration, and a decreased leptin concentration than those who had more than 5 h of sleep [15]. Second, sleep deprivation affects metabolism. The lack of sleep increases insulin resistance, which can increase the risk of developing obesity-related metabolic diseases [38]. Third, sleep deprivation affects energy intake [39]. Experiments showed that individuals with less than 4 h of sleep consumed more calories than did those who slept for longer, but the overall energy consumption did not differ [39]. Therefore, sleep deprivation leads to excessive caloric intake, a main mechanism of weight gain [39]. These studies show that sleep duration is important for lowering obesity risk. Sleep duration is also associated with sex, with men typically sleeping longer and experiencing less sleep disorders compared to women. This can be attributed to the fact that women often contend with higher rates of depression, fatigue, and various physical discomforts compared to men [40].

In particular, a study that examined the association between sleep duration and obesity based on sex revealed a distinct correlation, specifically indicating a higher likelihood of obesity among women with shorter sleep durations [41]. In the United States, a study analyzing data from the National Health and Nutrition Examination Survey I, which included a civilian, non-institutionalized population, also established a connection between inadequate sleep and obesity, particularly in women [42]. Moreover, a separate study conducted at an internal medicine clinic in the United States discovered a significant association between short sleep duration and obesity among American women aged 18 to 49 years [43]. Similarly, according to research from China, short sleep duration was linked to an increased risk of obesity, with a specific emphasis on women [44]. However, it is important to note that while these studies confirm the relationship between sleep duration and obesity in women, the mechanism behind this connection is complex. Studies examining sex-specific sleep patterns have shown that sleep health can be significantly impacted by various hormonal and physical changes occurring at different stages in a woman’s life, including puberty, pregnancy, and menopause [45]. Additionally, another study suggested that estrogen-mediated effects in women may contribute to obesity [46]. These prior investigations collectively highlight a stronger association between sleep duration and obesity among women, underscoring the need for further research in this area. In this study, the risk of obesity was low and statistically significant for >1 h and <2 h of CUS on the weekend, and the risk of obesity was lower for women when they slept for >1 h and <2 h than for men, though this difference was not statistically significant. Women had more obesity-related risk factors because their body composition has a relatively higher body fat percentage and lower muscle mass percentage than men. This supports the higher explanatory power for abdominal fat accumulation by the WHtR than BMI. Therefore, we can infer that women have a relatively higher propensity for sleep time and WHtR than men.

As per a prior study, an increased duration of weekend CUS, a lower BMI, and longer weekend sleep can collectively reduce the risk of obesity associated with sleep deprivation [16]. Hence, this study suggests that a consistently short duration of sleep during the week may be linked to an elevated risk of obesity in adults, highlighting the significance of adequate sleep as a crucial lifestyle factor in obesity prevention.

Additionally, the definition of obesity and its influence can vary depending on its measurement and the criteria applied. Most previous studies have used BMI as a proxy indicator for obesity to assess the risk of various obesity-related diseases among various indicators for evaluating obesity [4,16,23,47,48]. The use of WHtR as a measure of obesity has several advantages over other obesity indicators. The same criteria can be applied to other ethnic groups in international joint research, and this simple formula is easy for the general public and patients to understand [29]. However, few studies have examined the effects of sleep on obesity using WHtR indicators, and few studies have examined this according to sex. Until recently, among the indicators that measure obesity, the WHtR has been used for meta-analyses, suggesting that a large-scale prospective study of Korean individuals is required in the future to determine whether this indicator is suitable for Korean characteristics.

This study has some limitations. It analyzed the association between sleep duration difference and obesity during weekdays and weekends using KNHANES data and analyzed the indicators of obesity using the WHtR. However, as the KNHANES data consist of self-reported questionnaires, the actual sleep duration and reported sleep duration may differ. As this study was cross-sectional in nature, it is important to note that objective sleep duration and sleep quality should ideally be measured through clinical trials. This limitation restricts our ability to establish causality and determine the long-term effects of consistent sleep duration on obesity. While the results of this study do suggest an association between sleep duration and obesity based on sex, it is essential to acknowledge that this observed association might be influenced by unmeasured confounding factors. Moreover, given that typical sleep patterns vary across different demographic groups, future research should delve deeper into how sleep duration affects obesity based on specific demographic features. In addition, this study did not control for confounding variables such as dietary habits, food intake, and lifestyle factors related to food that influence obesity. Therefore, future research should incorporate supplementary studies that address variables associated with food. Additionally, it would be beneficial to validate the relationship between weekend CUS and obesity by stratifying teenagers into males and females. In light of these findings, there is a clear need for differentiated and efficient management strategies that take into account age-specific approaches and occupational characteristics to improve the sleep duration of adults. Efforts should focus on managing both physical and mental health to extend sleep duration effectively. Nonetheless, this study conducted in Korea highlights a significant discovery, namely, the association between sleep duration during weekdays and weekends and obesity. This revelation carries important implications, particularly for individuals who sleep less than 7 h a day. Weekend CUS could potentially serve as an innovative approach to preventing obesity. The outcomes of this study can serve as valuable foundational data for shaping policies aimed at enhancing the overall health of adults in Korea. It is imperative to garner continuous attention and implement a range of interventions to help adults realize that getting more than one additional hour of sleep on weekends can potentially reduce the risk of obesity.

## 5. Conclusions

This study examined the association between short weekday sleep and weekend CUS time in relation to obesity among Korean adults experiencing sleep deprivation. We defined obesity using the waist-to-height ratio rather than body mass index. We found that women had a lower risk of obesity than men when they engage in weekend CUS. Therefore, in situations where increasing weekday sleep duration is impossible due to sociocultural factors, incorporating additional sleep during the weekend through CUS may help reduce the risk of obesity. In addition, to improve the sleep time of adults, personalized and efficient management measures that take into account age-specific approaches and characteristics of one’s occupation are required, and efforts to extend sleep time by managing physical and mental health are needed.

## Figures and Tables

**Table 1:** General characteristics of the study population according to obesity.

Variables	Obesity			
	Yes (*n* = 3888)	No (*n* = 2902)	Total (*n* = 6790)	*p*-Value
	*n* (%)	*n* (%)	*n* (*%*)	
	CUS			≤0.0001
CUS ≤ 0	2222 (63.92%)	1254 (36.08%)	3476 (51.19%)	
0 < CUS < 1	192 (51.34%)	182 (48.66%)	374 (5.51%)	
1 ≤ CUS < 2	645 (50.35%)	636 (49.65%)	1281 (18.87%)	
CUS ≥ 2	829 (49.97%)	830 (50.03%)	1659 (24.43%)	
	Sex			≤0.0001
Men	2231 (61.6%)	1402 (38.6%)	3633 (53.51%)	
Women	1657 (52.5%)	1500 (47.5%)	3157 (46.49%)	
	Age (years)			≤0.0001
19–39	594 (37.2%)	1001 (62.8%)	1595 (23.49%)	
40–65	2544 (59.5%)	1733 (40.5%)	4277 (62.99%)	
>65	168 (18.3%)	750 (81.7%)	918 (13.52%)	
	Marital status			≤0.0001
Married	3504 (61.2%)	2225 (38.8%)	5729 (84.37%)	
Unmarried	384 (36.2%)	677 (63.8%)	1061 (15.63%)	
	House income			≤0.0001
Low	526 (73.5%)	190 (26.5%)	716 (10.56%)	
Mid-low	949 (63.0%)	557 (37.0%)	1506 (22.21%)	
Mid-high	1101 (53.9%)	941 (46.1%)	2042 (30.11%)	
High	1308 (52.0%)	1210 (48.1%)	2518 (37.13%)	
Missing data			8	
	Educational level			≤0.0001
Elementary school or below	757 (81.2%)	175 (18.8%)	932 (13.73%)	
Middle school	420 (68.6%)	192 (31.4%)	612 (9.01%)	
High school	1204 (55.7%)	957 (44.3%)	2161 (31.83%)	
College or above	1506 (48.8%)	1578 (51.2%)	3084 (45.43%)	
Missing data			1	
	Region			≤0.0001
Urban area	2996 (54.8%)	2468 (45.2%)	5464 (80.47%)	
Rural area	892 (67.3%)	434 (32.7%)	1326 (19.53%)	
	Occupation			≤0.0001
White collar	1403 (48.5%)	1490 (51.5%)	2893 (42.75%)	
Pink collar	1469 (63.5%)	845 (36.5%)	2314 (34.19%)	
Blue collar	1007 (64.5%)	554 (35.5%)	1561 (23.06%)	
Missing			22	
	Smoking status			
Non-smoker	1965 (53.9%)	1678 (46.1%)	3643 (53.72%)	≤0.0001
Ex-smoker	1086 (64.5%)	598 (35.5%)	1684 (24.83%)	
Current smoker	828 (57.0%)	626 (43.1%)	1454 (21.44%)	
Missing data			9	
	Alcohol consumption			≤0.0001
None	981 (64.9%)	531 (32.1%)	1512 (22.29%)	
≤1 time per week	1829 (52.1%)	1682 (47.9%)	3511 (51.77%)	
≥2 times per week	1070 (60.8%)	689 (39.2%)	1759 (25.94%)	
Missing data			8	
	Physical activity			≤0.0001
No	2230 (60.5%)	1459 (39.6%)	3689 (54.40%)	
Yes	1652 (53.4%)	1440 (46.6%)	3092 (45.60%)	
Missing data			9	

CUS, Catch-up sleep.

**Table 2:** Association between weekend catch-up sleep and obesity.

	Obesity			
	OR	95% CI		*p*-Value
Weekend CUS				0.3729
CUS ≤ 0	1.00			
0 < CUS < 1	0.87	0.69	1.09	
1 ≤ CUS < 2	0.86	0.75	0.99	
CUS ≥ 2	0.96	0.84	1.09	
Sex				<0.0001
Men	1.00			
Women	0.53	0.46	0.61	
Age (years)				<0.0001
19–39	1.00			
40–65	1.75	1.51	2.03	
>65	2.90	2.25	3.74	
Marital status				<0.0001
Married	1.00			
Unmarried	0.67	0.56	0.79	
House income				0.1045
Low	1.20	0.97	1.50	
Mid-low	1.14	0.98	1.31	
Mid-high	0.99	0.87	1.12	
High	1.00			
Education level				<0.0001
Elementary school or below	2.50	1.97	3.16	
Middle school	1.49	1.20	1.85	
High school	1.16	1.02	1.33	
College or above	1.00			
Region				0.0148
Urban area	1.00			
Rural area	1.19	1.04	1.37	
Occupation				0.0013
White collar	1.09	0.93	1.28	
Pink collar	1.31	1.12	1.52	
Blue collar	1.00			
Smoking status				0.0628
Non-smoker	1.00			
Ex-smoker	1.12	0.96	1.31	
Current smoker	0.94	0.80	1.10	
Alcohol consumption				0.0224
None	1.00			
≤1 time per week	0.86	0.75	0.98	
≥2 times per week	0.99	0.84	1.17	
Physical activity				0.0223
No	1.13	1.02	1.25	
Yes	1.00			

CUS, Catch-up sleep; OR, odds ratio; CI, confidence interval. Adjusted for age, sex, marital status, household income, educational level, occupation, region, smoking status, alcohol consumption, and physical activity.

## Data Availability

All data files are available from the Korea Centers for Disease Control and Prevention database through the following URLs: https://knhanes.cdc.go.kr/knhanes/sub03/sub03_02_05.do (accessed on 28 May 2023). However, the data access process and user manual are only written in Korean.

## Supplementary Materials

The following supporting information can be downloaded at: https://www.mdpi.com/article/10.3390/healthcare11212889/s1, Table S1: General characteristics of study population according to CUS.
