# Supplementary material for: Sex Differences Associated with Weekend Catch-Up Sleep and Waist-to-Height-Ratio among South Korean Adults Using Korea National Health and Nutrition Examination Survey 2016–2021 Data
Source: Healthcare (Basel). 2023 Nov 2;11(21):2889. doi: 10.3390/healthcare11212889 (PMC10648526; doi:10.3390/healthcare11212889)
Supplement: Supplementary file 1 [file healthcare-11-02889-s001.zip › healthcare-2657500-SI.pdf]

Table S1. General characteristics of study population according to CUS

Variable	WHtR(waist-to-height-ratio)						P-value	WHtR(waist-to-height-ratio)						P-value
	Male							Female						
	<0.5 (Normal)		≥0.5 (Obesity)		Total			<0.5 (Normal)		≥0.5 (Obesity)		Total		
	N	%	N	%	N	%		N	%	N	%	N	%	
Weekend-Catch up-Sleep							<.0001							<.0001
CUS ≤0	644	34.6	1218	65.4	1862	51.3		610	37.8	1004	62.2	1614	51.1	
0<CUS<1	256	4.7	373	59.3	629	17.3		319	54.0	272	46.0	591	18.7	
1≤CUS<2	313	43.9	400	56.1	713	19.6		339	58.9	237	41.2	576	18.3	
CUS≥2	189	44.1	240	55.9	429	11.8		232	61.7	144	38.3	376	11.9	
Age							<.0001							<.0001
19-39	486	52.0	449	48.0	935	25.7		515	78.0	145	22.0	660	20.9	
40-65	817	35.8	1468	64.3	2285	62.9		916	46.0	1076	54.0	1992	63.1	
>65	99	24.0	314	76.0	413	11.4		69	13.7	436	86.3	505	16.0	
Martial Status							<.0001							<.0001
Married	1077	35.6	1945	64.4	3022	83.2		1148	42.4	1559	57.6	2707	85.8	
UnMarried	325	53.2	286	46.8	611	16.8		352	78.2	98	21.8	450	14.3	
House income							0.0288							<.0001
Low	89	33.1	180	66.9	269	7.4		101	22.6	346	77.4	447	14.2	
Mid-low	289	35.9	516	64.1	805	22.2		268	38.2	433	61.8	701	22.2	
Mid-high	458	41.1	657	58.9	1115	30.7		483	52.1	444	47.9	927	29.4	
High	565	39.2	876	60.8	1441	39.7		345	59.9	432	40.1	1077	34.2	
Missing	3							5						

Education level							<.0001							<.0001
Elementary school or below	77	26.1	218	73.9	295	8.1		98	15.4	539	84.6	637	20.2	
Middle school	101	32.0	215	68.0	316	8.7		91	30.7	205	69.3	296	9.4	
High school	424	38.3	682	61.7	1106	30.4		533	50.5	522	49.5	1055	33.4	
College or above	800	41.8	1116	58.3	1916	52.7		778	66.6	390	33.4	1168	37.0	
Missing								1						
Region							<.0001							<.0001
Urban area	1183	40.2	1760	59.8	2943	81.0		1285	51.0	1236	4903.0	2521	79.9	
Rural area	219	31.7	471	68.3	690	19.0		215	33.8	421	66.2	636	20.2	
Occupation							0.1351							<.0001
White collar	668	40.2	995	59.8	1663	46.0		822	66.8	408	33.2	1230	39.0	
Pink collar	281	37.9	461	62.1	742	20.5		564	35.9	1008	64.1	1572	49.8	
Blue collar	442	36.6	767	63.4	1209	33.5		112	31.8	240	68.2	352	11.2	
Missing	19							3						
Smoking status							<.0001							0.102
Non-smoker	368	43.2	483	56.8	851	23.5		1310	46.9	1482	53.1	2792	88.6	
Ex-smoker	505	33.6	999	66.4	1504	41.4		93	57.7	87	48.3	180	5.7	
Current-smoker	529	41.5	745	58.5	1274	35.1		97	53.9	83	46.1	180	5.7	
Missing	4							5						
Alcohol consumption							<.0001							<.0001
None	178	33.5	354	66.5	532	14.7		353	36.0	627	64.0	980	31.1	
≤1 time per week	739	42.9	984	57.1	1723	47.5		943	52.7	845	47.3	1788	56.7	
≥2 time per week	485	35.3	890	64.7	1375	37.9		204	53.1	180	46.9	384	12.2	
Missing	3							5						
Physical activity							<.0001							<.0001
No	669	35.4	1223	64.6	1892	52.1		790	44.0	1007	56.0	1797	57.1	

Yes	732	42.1	1007	57.9	1739	47.9	708	52.3	645	47.7	1353	43.0
Missing	2						7					
Total	1402	38.6	2231	61.4	3633	100.0	1500	47.5	1657	52.5	3157	100.0
